# Chelators in the Treatment of Iron Accumulation in Parkinson's Disease

**DOI:** 10.1155/2012/983245

**Published:** 2012-06-13

**Authors:** Ross B. Mounsey, Peter Teismann

**Affiliations:** School of Medical Sciences, College of Life Sciences and Medicine Institute of Medical Sciences, University of Aberdeen, Foresterhill, Aberdeen AB25 2ZD, UK

## Abstract

Iron is an essential element in the metabolism of all cells. Elevated levels of the metal have been found in the brains of patients of numerous neurodegenerative disorders, including Parkinson's disease (PD). The pathogenesis of PD is largely unknown, although it is thought through studies with experimental models that oxidative stress and dysfunction of brain iron homeostasis, usually a tightly regulated process, play significant roles in the death of dopaminergic neurons. Accumulation of iron is present at affected neurons and associated microglia in the substantia nigra of PD patients. This additional free-iron has the capacity to generate reactive oxygen species, promote the aggregation of **α**-synuclein protein, and exacerbate or even cause neurodegeneration. There are various treatments aimed at reversing this pathologic increase in iron content, comprising both synthetic and natural iron chelators. These include established drugs, which have been used to treat other disorders related to iron accumulation. This paper will discuss how iron dysregulation occurs and the link between increased iron and oxidative stress in PD, including the mechanism by which these processes lead to cell death, before assessing the current pharmacotherapies aimed at restoring normal iron redox and new chelation strategies undergoing research.

## 1. Introduction

Parkinson's disease is a chronic, progressive disorder and the second most common neurodegenerative disease after Alzheimer's disease, with an overall prevalence in the general population of 0.3% [1]. The incidence of PD increases with age, making this the most important risk factor. The cardinal symptoms of tremor, rigidity and postural instability result from the loss of dopaminergic neurons in the substantia nigra pars compacta (SNpc) and its projections in the nigrostriatal tract [2]. Symptoms do not occur until dopamine levels have been reduced by 70-80%, meaning that neuroprotection is a crucial but difficult task. In most PD patients the disease is idiopathic: a combination of environmental factors and genetic susceptibility. Only 5% of cases are purely genetic, with a number of genes identified, serving a vital role in early onset forms of the disease. The majority of cases are multifactorial—a combination of factors including accumulation of toxic protein aggregates, oxidative stress and inflammation.

A further pathological feature of PD is the abnormal accumulation of iron at affected neurons. Iron plays a vital role in various physiological functions including DNA synthesis, mitochondrial respiration, and oxygen transport. Neuronally, iron is involved in myelination and neurotransmission and is the most abundant metal in the brain. Crucially in relation to PD, the metal acts as a cofactor for tyrosine hydroxylase (TH), the enzyme at the rate-limiting step in the synthesis of dopamine. The importance of iron in this process is underlined by *in vitro* studies showing that TH activity is stimulated dose dependently by iron [3]. While iron is important physiologically in these actions, in excess the metal can be toxic through oxidative stress. The locations of iron accumulation in neurological disorders mirror the regions affected by the relevant condition. This situation is maintained in PD as increased levels of iron have been found in the substantia nigra of PD patients [4–6] and subsequently implicated in numerous neurological disorders with parkinsonism symptoms [7]. It is not clear whether this is a cause of or a development secondary to neuronal degradation [8]. However, infusion of ferric iron into the SNpc can be used to create a model of dose-related, progressive parkinsonism including a reduction in dopaminergic activity [9]. This can be attenuated by treatment with the lazaroid U-74389G [10], showing that iron may play a prominent causative role in the death of neurons by oxidative stress and lipid peroxidation. It has also been found that chronic exposure (more than 20 years) to iron and other metals leads to an increased risk of developing the disease [11], again demonstrating that excess iron may, at least in part, induce PD pathogenesis.

Increased iron content is caused by a number of factors (reviewed in [7]), including a disturbed or “leaky” blood-brain barrier (BBB), occupational exposure [11] and disruption of the body's iron storage and transport mechanisms. Iron distribution and storage is normally tightly regulated in the body due to the deleterious effects that iron deficiency and, as relevant in this discussion, overload have. This complex homeostasis is maintained by the differential expression of proteins that regulate its cellular uptake, utilisation, and storage.

The access of iron to cells is controlled primarily by transferrin receptors; its storage by the protein ferritin and pigment melanin (reviewed in [12]), a by-product of dopamine oxidation. Iron binds to transferrin after carefully controlled absorption from the duodenum and circulates in the blood. It is taken into cells via transferrin receptors and stored in the centre of metalloproteins. Excess iron is stored as ferritin and lost when cells are shed in the gut. Stored iron is mobilised from hepatocytes and tissue macrophages in response to an acute need, with increased intestinal absorption requested when demand (primarily by erythroid cells for heme synthesis) exceeds the supply of stored iron [13]. The levels of ferritin are crucial: iron is relatively nontoxic when bound to ferritin but alterations in unbound (free) iron can cause problems, therefore ferritin levels must be closely regulated. At the posttranscriptional level, appropriate cellular iron storage is maintained by the iron regulatory proteins 1 and 2 (IRP1 and IRP2, resp.). When iron levels are low, IRPs bind to iron responsive elements (IREs) on the 3′- and 5′-untranslated regions of their respective mRNAs, thus inhibiting translation of ferritin RNA and thereby decreasing the iron-storage capacity and stimulating translation of the transferrin receptor mRNA, a glycoprotein which controls levels of free-iron. The system then works in the opposite direction once sufficient iron has been taken up to downregulate the process [14, 15], helping maintain a storage capacity relative to the level of iron and the body's current demands. The importance of ferritin has been demonstrated through overexpression of H (heavy chain) ferritin in dopaminergic neurons [16]. The second important storage protein is intimately related to nigral neurons. These neurons produce the dark pigment neuromelanin, which can bind heavy metals, particularly iron. Loss of melanised neurons is correlated with an abundance of nonheme iron (Fe^3+^) and a significant increase in redox activity, which is most pronounced in patients with the greatest loss of neuromelanised cells [17].

This change in redox state can contribute to oxidative stress and induce further cell death. This paper will summarise briefly the factors contributing to a dysregulation of iron in parkinsonian patients and its role in the disease pathology before discussing the methods aimed at restoring iron homeostasis.

## 2. Molecular Basis of Iron Dysregulation in PD

The full role of iron in the pathogenesis of PD has been frequently reviewed [18–20] with various interpretations placed on its significance. Iron toxicity occurs when the levels of iron exceed the binding capacity of transferrin leading to an excess of reactive, unbound iron in the body. It is then sequestered in cells where creating an overload can induce deleterious effects.

Iron exists in two forms in living organisms: its reduced form, Fe^2+^, and the oxidised Fe^3+^ state. The transfer between these states, from ferrous iron (Fe^2+^) to the ferric form (Fe^3+^) in a catalytic reaction with hydrogen peroxide (or molecular oxygen) known as the Fenton reaction, can yield the highly toxic hydroxyl radical (^•^OH) via the Haber Weiss reaction [21]. The Fenton reaction is a normal metabolic process, occurring at times including electron transfer in mitochondria and readily in the cytoplasm where a large proportion of iron is reduced. However, in PD patients the ratio of Fe^2+^ : Fe^3+^ is 1 : 3 rather than 1 : 1, as in control brains [22], although this figure can vary between sources [5]. The iron redox is, therefore, in favour of Fe^3+^. The presence of additional iron together with a diminished supply of antioxidants leads to an increased generation of hydroxyl radicals through various reactions in the microglia, producing a cascade of destructive events including oxidative stress, lipid peroxidation, and eventually apoptosis [23]. The iron increase in the pathology of PD is not dependent on systemic iron imbalance, with local dysregulation of the redox contributing to the overload in the respective tissues [13].

Under physiological conditions, the free radical species produced in this reaction are sequestered and inactivated by the body's army of antioxidants, including glutathione (GSH), superoxide dismutase, and catalase. However, a tip in the balance of the Fenton reaction due to excess ferrous iron means there is an overwhelming amount of free radicals produced, which disrupts the normal cellular redox state. The endogenous cellular defences (which are relatively low for the amount of oxygen the brain consumes) are compromised, and oxidative stress follows, triggering a cascade of deleterious effects throughout the cell. Depleted glutathione levels have been forwarded as an early factor in PD pathogenesis [24], as asymptomatic cases of the disease show a similar pattern of depletion as later in the disease [25]. But certainly, an increase in antioxidant production in combination with a compromised cellular defence system will lead to an increased vulnerability of these cells.

Maintaining iron redox through processes such as iron transport sequestration and release is tightly regulated [26]. Any deviation from the normal redox state can have serious consequences for cells and the organism as a whole due to the potential iron possesses to become toxic. Ferritin, capable of sequestering up to 4500 Fe^3+^ atoms [27] (although it is rarely saturated [28]), represents an important endogenous capacity to limit the presence of redox-active iron. The protein is primarily cytosolic and exists in two subunits with different tasks. The ferroxidase activity of the H-ferritin subunit converts harmful labile Fe^2+^ to relatively nontoxic Fe^3+^, thereby keeping it in this unreactive form [15, 29]. The L- (light chain) ferritin subunit stabilises this complex and promotes iron's long-term storage [27, 30, 31]. The direct impact of these components in the iron regulatory system can be evaluated when studying inherited disorders. Mutations in the gene coding for L-ferritin have been reported to cause a basal ganglia disorder similar to PD, highlighting the neurological consequences that excess iron can have [32].

Imbalance in regulation mechanisms [12] leads to an increased reactive free-iron pool. Immunohistochemistry studies show a 60% reduction in iron-transferrin receptor binding in PD [33], as well as a decrease in transferrin binding sites [34]. It has been found that in PD brains, excessive iron is not mirrored by an increase in ferritin levels [35, 36]—normally brain H-ferritin levels parallel the increased iron accumulation which occurs with age [37]. Furthermore, in dopamine neurons iron stored within the ferritin core can be reduced readily by products of dopamine oxidation such as superoxide and 6-OHDA, adding a further level of vulnerability to this neuronal population. In an aged individual, the ferritin can become heavily burdened with the excess iron, which accumulates with age. Within lysosomes, ferritin may become degraded releasing the bound iron and further increasing the level of reactive iron [38, 39]. Therefore, in PD the iron is unbound and free to initiate a range of cytotoxic and inflammatory effects, such as the activation of redox-sensitive transcriptional factor nuclear factor kappa-B (NF*κ*B) and cytokine release from activated microglia [40].

Oxidative stress is instrumental in PD pathogenesis, as confirmed by significant neurochemical, histological, biochemical, and physical evidence and as shown in several models, including 1-methyl-4-phenyl-1,2,3,6-tetrahydropyridine (MPTP) [41] and rotenone [42], which exert their neurotoxic effects by free-radical-mediated mitochondrial dysfunction and oxidative stress [43, 44]. MPTP has also been implicated in exacerbating iron-related biochemical abnormalities [45] through an increased expression of the divalent metal transporter 1 (DMT1) [46]. Oxidative stress is at a high basal level in the SNpc as the autoxidation of dopamine produces semiquinones, a toxic species themselves, which can also lead to the generation of reactive oxygen species [47] making dopaminergic neurons particularly vulnerable to iron excess and these subsequent effects. This level is augmented in PD, partly due to the presence of additional iron. An excess of iron may lead to a vast increase in the production of free radicals, which overwhelms the natural defensive mechanisms and causes damage at several cellular levels.

Oxidative stress may also impair the ubiquitin-proteasome system, thereby inhibiting cells' ability to clear degraded proteins. Iron released from neuromelanin increases oxidative stress in mitochondria, disrupting mitochondrial function and reducing the ATP-dependent proteasomal activity of 26S (the proteasome involved in the ubiquitin system)—effects which could be reversed in one study by superoxide dismutase and the iron chelator deferoxamine [48]. Failure of the proteasomal system to clear these proteins, which include excess *α*-synuclein, could lead to the formation of the proteinaceous inclusions Lewy bodies, the pathological hallmark of PD [2, 49], and dopamine-dependent neurotoxicity [50]. The metal has been implicated in the formation of these protein aggregates [51]. Along with other free radical generators (dopamine and hydrogen peroxide), iron promotes the aggregation of intracellular aggregates containing *α*-synuclein and ubiquitin in a human neuroblastoma cell line overexpressing A53T and A30P proteins [52].

The process of lipid peroxidation, another event upregulated in PD patients [53], may be induced and can propagate several effects that threaten cell viability. It can result in the production of 4-hydroxy-2-nonenol (HNE) [54], a highly reactive lipophilic *α*,*β*-alkenal capable of inducing apoptosis through a cascade of caspase activation [55], in addition to promoting DNA fragmentation and contributing to oxidative stress [56]. It has been found that lipid peroxidation can cause protein aggregation [18], producing Lewy bodies. Iron may constitute a link between the pathogenic events of oxidative damage and protein aggregation, with iron accumulating in Lewy bodies in PD [57] and promoting alpha-synuclein aggregation [58], an event reversed by the administration of an iron chelator [59].

**Figure 1:**
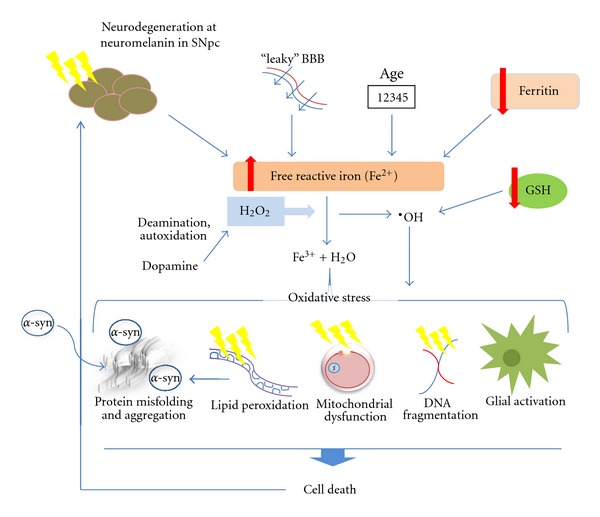
Iron-mediated cell death in PD. Reduced storage capacity in PD due to decreased ferritin expression and degeneration of nigral melatonin-containing neurons causes an increase in the reactive Fe^2+^ iron pool. Age-related increases in iron and a leaky BBB cause further iron accumulation. The transfer of the free iron to ferric iron, Fe^3+^, in the hydrogen peroxide-mediated Fenton reaction produces the highly toxic hydroxyl radical. A compromised level of glutathione exacerbates the levels of free radicals, whilst the deamination and autoxidation of dopamine produces further H_2_O_2_. The subsequent oxidative stress can then elicit a range of cytotoxic reactions including protein misfolding, lipid peroxidation (which, in turn, can cause *α*-synuclein aggregation), mitochondrial dysfunction, and activation of glial cells. These various insults can induce cell death by apoptosis, causing further degeneration.

It is, therefore, the proficiency of unbound iron to generate free radicals and induce oxidative stress, which is at the centre of their deleterious effects. This relationship is summarised in Figure 1. An imbalance in the normal redox state of iron, due to an age-related accumulation of iron in combination with dysregulation of the metal's storage, transport, and excretion systems, lead to increased levels of ferrous iron, which produce free radicals that overwhelm endogenous cell defences and generate a cascade of cytotoxic effects. The intimacy of iron storage sites in neuromelanin to dopaminergic neurons in the substantia nigra mean these cells are particularly at risk of iron overload-induced death.

## 3. Iron Chelators as a Treatment

**Figure 2:**
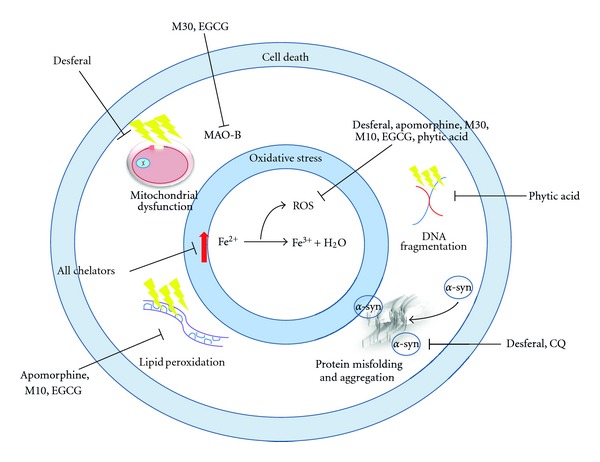
Action of iron chelators targeting PD. All iron chelators mop up excess free, reactive iron, thus reducing the reduction of Fe^2+^ to Fe^3+^—a reaction that produces various ROS, such as the hydroxyl radical. Oxidative stress resulting from the generation of ROS produces a range of deleterious insults, which can be targeted with the multiple actions of inhibitors. This can attenuate the cell death that these events induce. Chelators with antioxidant properties inhibit the production of ROS, in an environment of diminished antioxidant activity. The dopamine-oxidising enzyme MAO-B, which resides in the outer membrane of mitochondria, can also be inhibited by some chelators.

The correlation between iron accumulation in the brain and PD has logically led to the theory that chelators of iron could help slow the development of the disease by mopping up the unbound, free radical-enhancing iron in the brain. This mode of treatment has precedent: copper chelation using D-penicillamine has been effective in the removal of neuronal copper in Wilson's disease [60]. The treatment of iron dysregulation in PD at least holds the advantage over Alzheimer's disease (AD) that only one metal need be targeted, whereas with AD copper and zinc have also been associated with the disease pathology. Moreover, chelation of copper using D-penicillamine is ineffective against MPTP-induced dopamine depletion in mice [61]. Clear guidelines exist on the design and structure of a suitable iron chelator [62]. Potential chelators must have the ability to selectively scavenge excess intracellular iron and turn into a nontoxic product, which can be safely excreted. Since chelation therapy would be maintained for life, it is important that the agent does not impinge upon levels of other metals taken in by the diet. Chelators should only access the intracellular reactive labile iron pool, that is, iron that is not bound in a ferritin-iron complex as this is essential for normal physiological functions. The need for chelators to readily cross the BBB means that the size of potential chelators is important—300 Da being the stated maximum [63]. There are further substances that have found neuroprotective benefits in conditions of iron accumulation, but this is often due primarily to their antioxidant properties and/or by increasing mitochondrial activity. This paper will discuss those with proven iron chelating abilities, which often work in combination with some other neuroprotective abilities.  Table 1 summarises the information outlined in the following sections, with the modes of action of the chelators shown in Figure 2.

### 3.1. Chemical Chelators

Chemical iron chelators are already available clinically for various conditions. The use of them in neurodegenerative disorders has been restricted primarily by their ability (or lack thereof) to cross the BBB in therapeutically efficacious concentrations. But steps have been taken to overcome these limitations, which has produced a range of effective drugs to address iron overload in PD by various means.

### 3.2. Desferal

Desferrioxamine (also known as deferoxamine, DFO, or desferal) is a cell-impermeable, highly selective chelator, which can be taken up by the cell through endocytosis. It is a hexadentate iron chelator (i.e., it binds all six of iron's electrochemical coordination sites) and has been the most widely used iron chelator over the last 30 years [78], having been developed for the treatment of secondary iron overload (such as in *β*-thalassemia [79]), with the target primarily being the excess iron present in the liver and spleen. Desferal has also been used successfully to chelate iron in cases of aceruloplasminaemia, as assessed by MRI imaging, which parallels with improved neurological function [80]. However, this chelator has been unsuccessful previously at removing small increases in iron, such as in the joints of rheumatoid arthritis patients, due to adverse effects, which have been attributed to the high doses used [81]. Some factors make it unclear whether desferal would be suitable to treat excessive iron related to neurodegenerative diseases namely, whether it is able to remove excess iron without interfering with normal iron metabolism and if these small, hydrophobic molecules can cross the BBB.

Desferal is reported to attenuate iron-induced oxidative stress and mitochondrial dysfunction and prevent *α*-synuclein aggregation in the human neuroblastoma cell line SK-N-SH in culture [82]. Desferal was effective against MPP^+^-induced toxicity in a microdialysis study [83]. The drug also provided neuroprotection *in vivo* in a concentration-dependent manner in the 6-OHDA model of PD [22, 84]. The drug has been effective against dopaminergic neuron loss in rats [85] and in MPTP-treated mice [86] with iron overload. Desferal has been shown to attenuate nigral cell death induced by lactacystin [87]. However, the high hydrophobic nature and subsequent poor BBB permeability of desferal means that in these *in vivo* studies it was delivered by intracerebroventricular (ICV) injection—clearly not a viable option for clinical use. Therefore, effective chelators that can be delivered peripherally and effect iron levels in the brain are required. More recently, systemic administration of desferal at 30 mg/kg has been shown to be neuroprotective in the 6-hydroxydopamine (6-OHDA) model of PD and reduced hydroxyl radical formation [64]. There has been much progress delineating the mechanism and safe doses of desferal. However, the difficulty of administering the drug clinically limits its use sharply.

### 3.3. Deferiprone

The chelator deferiprone, used in the treatment of thalassaemia major, has the great advantage of being orally active; meaning the complex and mostly impractical administration routes of desferal may be avoided. It has been reported to be successful after 6 months of therapy in one case of neurodegeneration associated with brain iron overload, with an improvement of gait and reduction in dyskinesias [65], demonstrating that the drug can cross the BBB in efficacious concentrations. This led to the development of a Phase II trial for deferiprone to assess its safety and efficacy as a chelator of excess iron in the treatment for PD, with low doses being favoured (15 mg/kg/day). Patients are to be assessed every 3 months by unified PD rating scale (UPDRS) and, when possible, by magnetic resonance imaging [88]. Further clinical trials are assessing deferiprone at different doses in aged individuals [89, 90]. A smaller Phase II pilot study has already been completed, assessing the safety and efficacy of deferiprone on targeting globus pallidus iron in patients affected by pantothenate kinase-associated neurodegeneration (PKAN), a genetic disorder where levels of cysteine, an efficient iron binder, increase in the basal ganglia. The study, which nine patients completed, shows that 6 months of treatment leads to a significant reduction in iron content in this brain region, as measured by MRI, although the clinical status of patients did not change [66]. However, the patients in the study were not aged (the range being 7–39 years old), so whether deferiprone would be equally well tolerated in older PD patients is not certain. But the clear efficacy in reducing iron content in this form of neurodegeneration with brain iron accumulation (NBIA) is promising and may be well transferred to PD patients. Earlier intervention could be required to provide a symptomatic benefit.

### 3.4. Apomorphine

Pretreatment with R-apomorphine, a D1- and D2-receptor agonist, which can be used as a Levodopa replacement therapy in late-stage PD, shows antioxidant and iron-chelating abilities in the MPTP model due to its catechol structure [91]. The authors report that the dopamine agonist property is not behind the neuroprotective ability of the drug in this model, with a combination of iron chelating and, primarily, radical scavenging capacities accounting for the protection afforded [92]. This is because the nondopaminergic receptor agonist isomer of the drug, S-apomorphine, retains the same neuroprotective properties [93]. A broad spectrum of neuroprotective abilities is shown again by R-apomorphine *in vitro* [94]. These studies confirm that both inhibit the reduction in GSH, again confirming the benefit of a drug with multiple neuroprotective actions. The comprehensive assessment of R-apomorphine by [67] demonstrates the drug's neuroprotective actions against 6-OHDA- and iron-induced toxicity *in vitro* as well as in MPTP mouse models. These include inhibition of iron-mediated lipid peroxidation, meaning this agent elicits an extensive range of actions to counter the damage caused by iron accumulation.

### 3.5. Hydroxyquinolines

Hydroxyquinoline is a bidentate chelator containing the 8-hydroxyquinoline (8-HQ) moiety. This structure forms stable 5-membered chelate rings with Fe^3+^ [95]. 8-HQ, a highly lipophilic complex, readily penetrates cell membranes and the BBB and, therefore, has been used as the base for orally effective chelators, including VK-28, M30, and clioquinol, all of which will be discussed.

VK-28 is an iron chelator with a potency comparable to desferal. However, in contrast to desferal, VK-28 is BBB-permeable. It is neuroprotective against 6-OHDA toxicity in rats when given either by ICV or intraperitoneal routes without altering peripheral iron metabolism. In the absence of the toxin, VK-28 has no effect on the basal levels of transmitters such as dopamine, showing that the iron-dependent rate-limiting enzymes TH and tryptophan hydroxylase are not affected [68].

Since VK-28 so far meets all the needs of an iron chelator, derivatives of VK-28 have been generated. M30, a potent brain-selective inhibitor of monoamine oxidase-A and -B (MAO-A and MAO-B, resp.) based around the pharmacore of VK-28, is effective in both cell culture [70] and the MPTP mouse model [69]. It also restores levels of the antiapoptotic protein Bcl-2 in mice treated with microinjections of lactacystin [71]. Both M30 and parent molecule VK28 showed behavioural improvements in this model [70]. M30 also prevents iron-dependent hydroxyl radical generation [96]. Part of this bifunctional protection is explained by the drug sharing some structural units with rasagiline, a selective irreversible MAO-B inhibitor [97]. M10 is another hydroxyquinoline derivative with radical scavenging and iron-chelating properties. It is a potent hydroxide scavenger and has been shown to be as effective as rasagiline in PC10 cell culture and inhibits lipid peroxidation with an IC50 value comparable to desferal [96].

Antibiotics have been shown to have iron-chelator properties. 5-chloro-7-iodo-hydroxyquinoline (or clioquinol/CQ) was at the centre of one of the worst drug disasters of the 20th century, when thousands of people worldwide developed subacute myelo-optic neuropathy (SMON) after using the drug to treat intestinal infections. This pathology was later linked to the drug's promotion of vitamin B12 excretion [72]. It has long been known that clioquinol chelates iron [98]. More recently it has been studied for its iron-chelating properties in neurodegenerative diseases. CQ has been reported to be effective against *β*-amyloid aggregation in AD transgenic mice [99] in a study targeting copper and zinc interactions, showing that CQ is not a selective iron chelator. However, despite this apparent drawback when considering the guidelines outlined [78], the converse was the case, as copper can also facilitate Fe^2+^ toxicity [99]. Toxic side effects have been a problem when using other iron chelators; the low lipid solubility of which hinders their ability to cross the BBB meaning higher doses are required. The lipophilic nature of CQ allows for its effective use at lower concentrations [72, 73]. CQ provided neuroprotection in MPTP-treated mice [73] after 8 weeks of oral treatment in addition to a reduction in motor dysfunction. CQ led to decreased bioavailable iron levels in normal mice [72], with the drug well tolerated in both studies, an important consideration to make since any clinical treatment is likely to be for life.

### 3.6. Natural Chelators

This group includes components of plant polyphenols, such as epigallocatechin gallate (EGCG), a green tea extract (catechin). These compounds have been utilised over many years for their antioxidant properties and have been investigated for their potential use in several diverse areas including oncology, cardiology, and neurology. The favourable properties of green tea (GT) are attributed to their high content of antioxidant polyphenic flavanoids, of which there is a vast array—over 4000 flavanoids have been identified [100]. GT catechins possess structures which infer metal chelating properties—the 3′,4′-dihydroxyl group and the gallate groups are present. These may neutralise ferric iron to produce a redox-inactive form [101]. The compounds are nontoxic and readily cross the BBB, meaning the ease of administration is a major advantage, with epidemiological evidence showing that drinking two cups of green tea a day reduces the risk of PD [102].

### 3.7. EGCG

The most abundant and pharmacologically active green tea extract, EGCG, has been shown to be attenuate striatal dopamine and TH loss as well as nigral dopaminergic cell death in MPTP-treated mice [103]. The authors propose various mechanisms for this protection including acting as an antioxidant, preventing lipid peroxidation and inhibiting MAO-B in addition to its role as an iron chelator. An agent which could work by various pathways would be highly beneficial due to the multifactorial nature of PD. Catechins have also been shown to regulate various signalling pathways involved in cellular survival and downregulate proapoptotic pathways (reviewed in [104]), as well as inhibit catechol-O-methyltransferase (COMT) activity [105]. Together with its iron-chelating activities, this data mean green tea extracts are powerful agents to use when cell death may be mediated by oxidative stress.

EGCG has been tested in combination with the established anti-PD drug rasagiline [74]. EGCG is relatively a far weaker MAO-B inhibitor than rasagiline (with an IC_50_ of 662 *μ*M compared to 6 nM [103]); therefore, if this pharmacological requirement could be supplemented by another drug, the neuroprotective effect may be augmented. It was found that the two act synergistically to provide neuroprotection in the MPTP mouse model [74].

However, EGCG has proven less successful in the 6-OHDA rat model as only subtle behavioural improvements in the absence of neuroprotective benefits were produced when given orally [75]. This may be due to the doses administered (1-2 mg/kg: physiological dose), which, although effective against MPTP-induced neuronal degradation [103], were too low for this particular model due to the mechanism of toxicity. Therefore, it is important to consider the actions of individual PD models when evaluating the effectiveness of these compounds.

### 3.8. Phytic Acid

Phytic acid (IP6, myoinositol hexakisphosphate) is an antinutrient and has previously been shown to be effective in cancer through an antioxidant effect [106]. The agent has been studied for its beneficial antioxidant properties on hydroxyl radical formation enhanced by Fe^2+^ induced by MPP^+^ in rat striata. Here phytic acid chelated iron required for radical formation via the Fenton reaction [107]. It has since been demonstrated to be protective against MPP^+^-induced toxicity in immortalised rat mesencephalic/dopaminergic cells by attenuating caspase-3 activity and DNA fragmentation, and increasing cell viability in both conditions of normal and excess intracellular iron content [76]. This effect was replicated in a cell culture model utilising 6-OHDA [77]. Phytic acid has been forwarded as a safer alternative to synthetic iron chelators, such as desferal, although there remains some concern about the compound's ability to cross the BBB. Studies in PD models are also limited at this stage. Further work is required with this compound to fully assess its potential clinical benefit.

These groups of natural chelators meet all the requirements for an iron chelator to be effective in providing neuroprotection in PD. The range and vast number of compounds available mean there are many more similar structures which can be screened. Further tests in models of PD would allow them to be effectively evaluated.

## 4. Conclusions

There is strong evidence that iron accumulation causes cell death, particularly in the substantia nigra through oxidative stress mechanisms. Deposits of the metal are found in the substantia nigra of PD patients, whilst local administration of the metal can cause degeneration and reductions in dopamine levels. Iron is involved in various biochemical reactions, with the Fenton reaction at the centre of this redox balance. The transfer between the two ion states is a tightly regulated process, in which storage and transfer proteins work concurrently to maintain physiological levels of iron. An influx of the metal causes an increase in the levels of reactive ferric iron and results in more harmful reactive species. Deleterious consequences including lipid peroxidation, reduction in levels of endogenous antioxidants and stimulation of cellular apoptotic cascades escalate cell death in these areas. Iron chelators have the potential to address the balance in the available level of the reactive iron form (labile iron pool) to prevent the overproduction of oxidative species.

 Established iron chelators, which have been used in conditions of iron excess for many years, such as desferrioxamine/desferal, have now found a new use in neurodegenerative diseases. But desferal's inability to cross the BBB means this drug will never be a feasible option in its initial form. However, the drug's use as a base for orally active agents, which retain these iron chelating activities, is more significant. Of the putative treatments, deferiprone is certainly at the most advanced levels of research. Having found success in experimental models, the recent commissioning of Phase II clinical trials investigating the agent will provide further information regarding the safety and tolerability in aged patients.

 A major advantage of these new pharmacotherapies is the mixture of neuroprotective actions they possess. In addition to iron chelation, recent therapies also have MAO-B inhibition capabilities and antioxidant properties. This allows for a more comprehensive mode of action, which is what is required in PD where a plethora of pathologies exist. Antioxidant actions of natural substances, such as the green tea extract EGCG, have been coupled with iron chelation activities to provide natural alternatives.

However, due to the ubiquitous nature of iron, it is important to consider whether iron chelators will provide the cellular specificity required to remove excess iron from the appropriate tissue, without affecting systemic iron homeostasis. The dose of chelator is an important factor to consider in treatment so that the correct tissues are penetrated and systemic iron levels are not altered. Moreover, subcellular locations of iron may need to be recognised. Li and colleagues [108] discuss the importance of specificity of intracellular targets for iron chelation in relation to Friedreich's ataxia (FA). Iron chelators, therefore, may be required to remove iron in a highly selective manner whilst stabilising iron levels elsewhere even within the same cell. Initial clinical trials [65] demonstrate that doses of deferiprone are well tolerated over the long term (6 months) with no stated propagation of a challenge to systemic iron homeostasis. The early successes of these patient studies demonstrate that this issue may not be as pertinent in PD as in FA. Furthermore, deferiprone itself has been shown to be highly selective when used at low concentrations in FA patients—relocating iron from areas of accumulation in the brain to ferritin, thereby preventing deprivation in other tissues [109].

The presence of increased levels of iron in the areas affected by degeneration in PD together with the effects this dysregulation have, mean reversing this redox imbalance is an important aim for any neuroprotective therapy. Iron chelators initially used for other medical applications have been built upon with their functions adapted to suit the pathology of PD. With some treatments now reaching clinical trials, it is hoped that an efficacious and tolerable iron chelation therapy, which is effective at attenuating neurodegeneration, is close to being achieved.

## Figures and Tables

**Table 1:** Iron chelators in brief. Summary of key information regarding iron chelators currently undergoing research as possible PD therapies.

Chelator name	BBB-permeable	Stage of research	Relevant findings	References
Synthetic				
Desferal	No	Clinically used for systemic iron accumulation. Cellular and animal models of PD	Neuroprotective in rat 6-OHDA model	[64]
Deferiprone	Yes	Phase II trials	Efficacious. Can reduce iron levels but not always with symptomatic improvement	[65, 66]
Apomorphine	Yes	Animal models	Effective against iron-induced toxicity and MPTP-induced cell death	[67]
VK-28	Yes	Animal models	Protective in 6-OHDA rat model	[68]
M30	Yes	Animals models	MAO-A and -B inhibitor. Selective. Effective in MPTP mouse model	[69]
M10	Yes	Cell culture	Hydroxide scavenger. Inhibits lipid peroxidation	[70, 71]
CQ	Yes	Animal models	Neuroprotective in MPTP mouse model	[72, 73]

Natural				
EGCG	Yes	Animal models	Multiple protective actions. Can be used in combination with rasagiline. Effective in MPTP mouse model but not in 6-OHDA rat model	[74, 75]
Phytic acid	Unknown	Cell culture	Protects against MPP^+^ and 6-OHDA toxicity in normal and excess iron	[76, 77]
